# Analysis of avian Usutu virus infections in Germany from 2011 to 2018 with focus on dsRNA detection to demonstrate viral infections

**DOI:** 10.1038/s41598-021-03638-5

**Published:** 2021-12-17

**Authors:** Theresa Störk, Madeleine de le Roi, Ann-Kathrin Haverkamp, Sonja T. Jesse, Martin Peters, Christine Fast, Katharina M. Gregor, Laura Könenkamp, Imke Steffen, Martin Ludlow, Andreas Beineke, Florian Hansmann, Peter Wohlsein, Albert D. M. E. Osterhaus, Wolfgang Baumgärtner

**Affiliations:** 1 Department of Pathology, University of Veterinary Medicine Hanover Foundation, Bünteweg 17, 30559 Hannover, Germany; 2 Research Center for Emerging Infections and Zoonoses, University of Veterinary Medicine Hanover, Hanover, Germany; 3 Chemisches Und Veterinäruntersuchungsamt Westfalen, Arnsberg, Germany; 4 Friedrich-Loeffler-Institut, Federal Research Institute for Animal Health, Institute of Novel and Emerging Infectious Diseases, Greifswald-Insel Riems, Germany; 5 Institute for Biochemistry, University of Veterinary Medicine Hanover, Hanover, Germany; 6 Institute of Veterinary-Pathology, Leipzig University, Leipzig, Germany

**Keywords:** Zoology, Diseases, Medical research

## Abstract

Usutu virus (USUV) is a zoonotic arbovirus causing avian mass mortalities. The first outbreak in North-Western Germany occurred in 2018. This retrospective analysis focused on combining virological and pathological findings in birds and immunohistochemistry. 25 common blackbirds, one great grey owl, and one kingfisher collected from 2011 to 2018 and positive for USUV by qRT-PCR were investigated. Macroscopically, most USUV infected birds showed splenomegaly and hepatomegaly. Histopathological lesions included necrosis and lymphohistiocytic inflammation within spleen, Bursa fabricii, liver, heart, brain, lung and intestine. Immunohistochemistry revealed USUV antigen positive cells in heart, spleen, pancreas, lung, brain, proventriculus/gizzard, Bursa fabricii, kidney, intestine, skeletal muscle, and liver. Analysis of viral genome allocated the virus to Europe 3 or Africa 2 lineage. This study investigated whether immunohistochemical detection of double-stranded ribonucleic acid (dsRNA) serves as an alternative tool to detect viral intermediates. Tissue samples of six animals with confirmed USUV infection by qRT-PCR but lacking viral antigen in liver and spleen, were further examined immunohistochemically. Two animals exhibited a positive signal for dsRNA. This could indicate either an early state of infection without sufficient formation of virus translation products, occurrence of another concurrent virus infection or endogenous dsRNA not related to infectious pathogens and should be investigated in more detail in future studies.

## Introduction

In summer of 2018, a mass mortality of common blackbirds (*Turdus merula*) was observed in Germany, for which Usutu virus (USUV) was identified as the causative agent^1^. This mosquito-borne flavivirus belongs to the Japanese encephalitis virus serogroup and is closely related to West Nile virus (WNV) and Japanese encephalitis virus (JEV). USUV is named after the Usutu river in Swaziland (Africa), where it was detected for the first time in *Culex neavei* mosquitoes in 1959^2^. In the following years, USUV spread not only across Africa^3^ and across the Mediterranean basin, but was repeatedly introduced into Europe via migratory birds^4^.

The first detection of USUV in Europe was dated to 1996 in a retrospective study of dead blackbirds in Italy^5,6^. In 2001, USUV infections caused mass mortalities in common blackbirds, great grey owls (*Strix nebulosa*), and barn swallows (*Hirundo rustica*) in Vienna in Austria^7^. Nine years later, USUV was detected for the first time in *Culex pipiens* mosquitoes in Germany^8^. In the subsequent summer of 2011, an USUV related mass mortality occurred in wild and zoo birds in South-West Germany^9^. While relatively low numbers of lethal USUV cases have been reported in birds in South-West Germany between 2011 and 2014^4,10^, fatal cases occurred in great grey owls in a zoological garden in Berlin with introduction of a new USUV lineage in 2015^11^. In 2016, further spread of USUV to Western neighboring countries including Belgium, France and the Netherlands was detected^12^. Thus, similar outbreaks arose in France^13^ and the Netherlands^14^ in 2015 and 2016, respectively. Lastly, 15,000 dead birds were reported all across Germany in summer of 2018, although the number of unreported cases is suggested to be much higher. Most of the affected birds were common blackbirds but also a high number of migratory birds was affected. In addition, blue tits (*Cyanistes caeruleus*), green finches (*Chloris chloris*), house sparrows (*Passer domesticus*), thrushes (*Turdus philomelos*), robins (*Erithacus rubecula*), and great tits (*Parus major*) were affected (https://www.nabu.de/tiere-und-pflanzen/voegel/gefaehrdungen/krankheiten/usutu/26311.html, accessed March 2nd 2020).

Similarly to WNV, USUV is maintained and transmitted by a vertebrate host-mosquito sylvatic life cycle in which mosquitoes act as vectors and birds as the main amplifying hosts^15^. In Europe, *Culex pipiens* and *Aedes albopictus* in particular have been identified as vectors^16–18^. However, it is known that other mosquitoes, such as *Ochlerotatus sp.* or *Anopheles sp.* can also be carriers of USUV^16,17^. Until today, USUV has been detected in 93 different avian species with common blackbirds being by far the most frequently affected species^19^. However, great grey owls and other birds of prey are also highly susceptible to USUV infection^17^. Macroscopically, affected birds show predominantly hepatomegaly, splenomegaly, an empty gastrointestinal tract or mucoid enteritis^11,20,21^. Histologically, they mainly exhibit necrotizing hepatitis and splenitis. In addition, myocardial degeneration and/or myocarditis as well as neuronal necrosis, neuronophagia and gliosis have been described in previous reports^7,20–22^. Other organs occasionally affected by either necrosis and/or lymphohistiocytic or lymphoplasmacytic infiltrates include kidney, lung, proventriculus, intestine, pancreas, mesenteric fat tissue and skeletal muscle^11,21^.

Non-avian species like horses^23^, dogs^24,25^, wild boars^26^, wild ruminants^27^, rodents^28^, squirrels^29^, and bats^30^ can serve as accidental hosts but infection in these species is often asymptomatic. It is uncertain whether some of these species can act as secondary reservoirs allowing establishment of viremia for infection of new mosquitoes^31^. In humans, antibodies have been detected in healthy people^32–34^ with the highest seroprevalence being identified in serum samples from asymptomatic forestry workers in Italy^35^. Moreover, fever and rash have been observed in patients^3^ and life-threatening meningoencephalitis and peripheral nerve paralysis occur in a low number of humans, highlighting USUV as a potential threat to human health^32,36–39^. This underlines the importance of arbovirus surveillance programs and the need for more precise descriptions of pathological lesions and pathogenesis.

Nonetheless, surveillance systems and detection of new arising or previously unrecognized viruses are challenging, because most conventional methods rely on the detection of virus-specific antigens or nucleotide sequences^40,41^. In contrast, molecular analysis, including Next Generation Sequencing (NGS), represents a sequence-independent but also cost-intensive approach^42–44^. Detection of double-stranded ribonucleic acid (dsRNA) by immunohistochemistry is expected to be a sensing tool for future investigation of etiologically unknown or new emerging virus infections^45^. The usability of dsRNA as an alternative approach for virus detection was first studied in virus infections in plants^46,47^. Later studies investigated the occurrence of dsRNA in viral infections caused by *Flaviviridae*, including WNV and Dengue virus^48,49^. DsRNA can be found during viral infections regardless of the constitution of viral genome^50^. In single-stranded, positively orientated RNA virus infections, including USUV, dsRNA is produced as a replicative intermediate during virus replication^41,49–51^. DsRNA represents also a key factor for immune response induction in both mammalian and avian cells^52,53^. Recognition of dsRNA is mediated by pattern recognition receptors which sense cytosolic dsRNA among numerous other pathogen-associated molecular patterns and initiate signaling pathways that lead to an activation of the innate immune system aiming to trigger an antiviral response^54^.

Since USUV infection was rather uncommon in Northern and Western Germany prior to 2018, it was the aim of the present study to (a) characterize the currently circulating viral variants, (b) elucidate lesions commonly associated with USUV infection in birds, (c) clarify the distribution of viral antigen, (d) compare lesions and virus distribution with reports of previous outbreaks in and outside Germany, and foremost (e) evaluate the role of alternative detection methods by using dsRNA-specific antibodies as a proof-of-principle sensing tool for USUV and as a potential sensing approach for future outbreaks of viral diseases of unknown etiology.

## Results

### Anamnesis and macroscopic findings

In this study, organs of 25 common blackbirds, one great grey owl, and one kingfisher, collected over a period from 2011 to 2018 and tested positive for USUV and negative for WNV by quantitative real-time PCR (qRT-PCR) at the Friedrich-Loeffler-Institut (FLI) on Riems in Germany, were investigated. Prior apathy was reported in two animals (7%; 2/27; Supplementary Table S1, #1, #2). They died one day after onset of clinical signs. A third animal (4%; 1/27; Supplementary Table S1, #21) died following trauma and in all remaining cases (93%; 25/27), birds were found dead without additional information (Supplementary Table S1).

**Figure 1:**
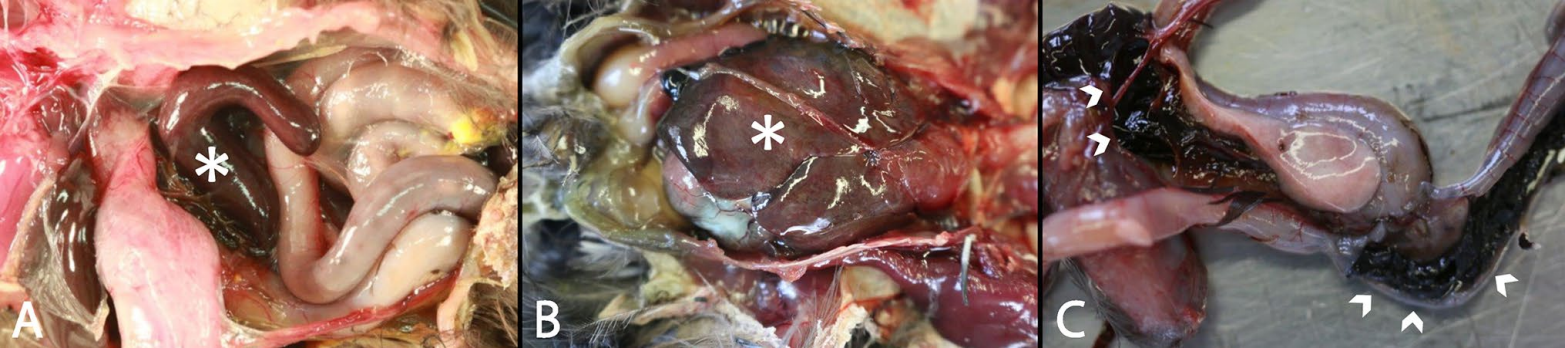
Macroscopic findings in naturally Usutu virus infected birds. (**A**) Marked splenomegaly (asterisk) was present in a blackbird (Supplementary Table S1, #8). (**B**) Moderate to marked hepatomegaly (asterisk) was shown in a blackbird (Supplementary Table S1, #10). (**C**) Severe hemorrhagic enteritis characterized by dark red to black, coagulated blood in the intestinal lumen was detected in a blackbird (arrowheads; Supplementary Table S1, #8).

Macroscopic examination revealed splenomegaly (71%; 12/17; Fig. 1A), hepatomegaly (35%; 6/17; Fig. 1B), massive hyperemia of different internal organs (24%; 4/17), and/or subcutaneous edema (12%; 2/17; Supplementary Table S1, #1, #3). Additionally, hemorrhagic enteritis was noticed in one animal (6%; 1/17; Fig. 1C; Supplementary Table S1, #8). However, some animals displayed no significant macroscopic findings (18%; 3/17) or were not examined macroscopically (41%; 11/27; Supplementary Table S1).

### Histopathology

**Figure 2:**
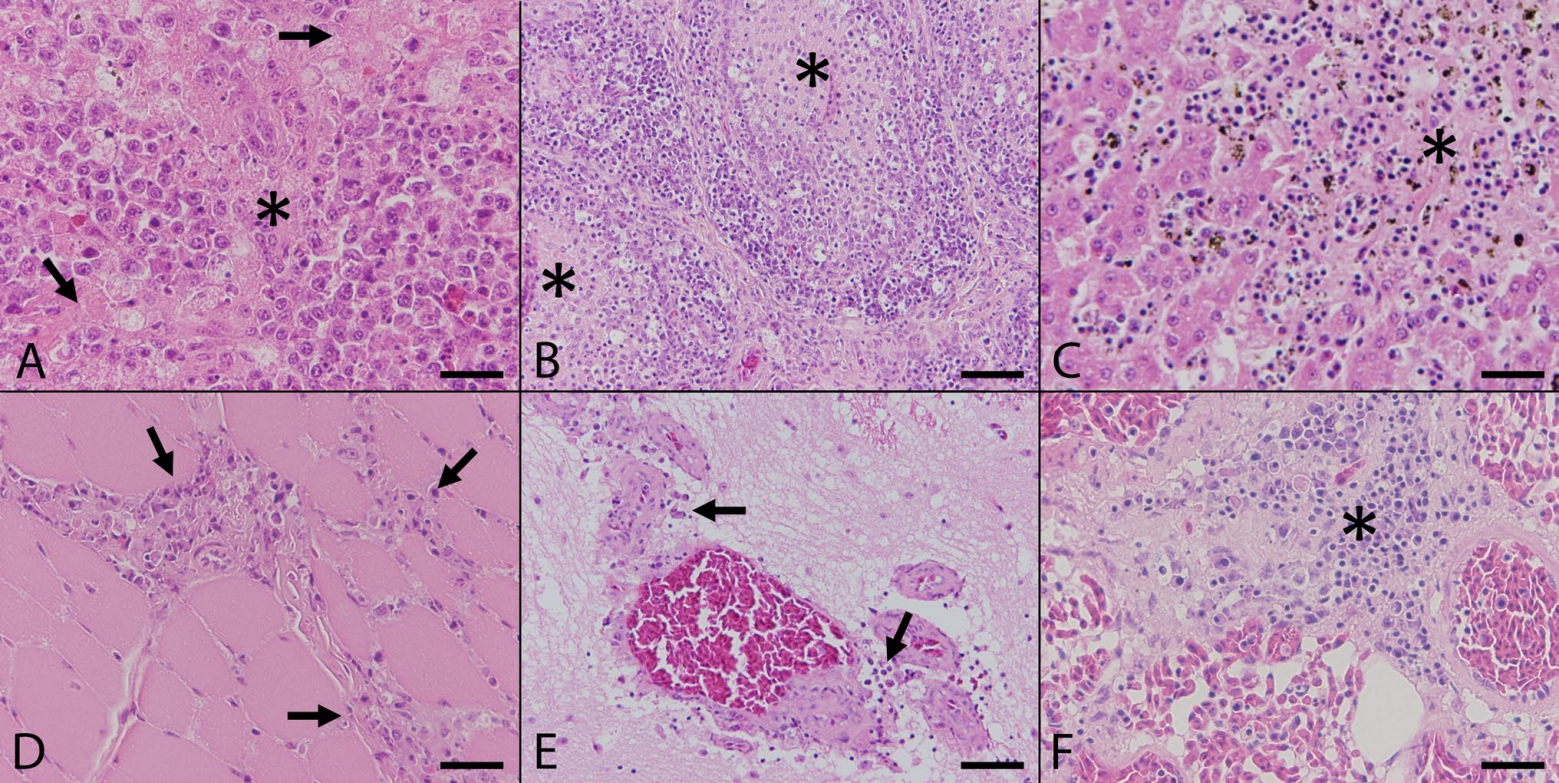
Histopathologic findings in naturally Usutu virus infected birds. (**A**) The Spleen of a great grey owl (Supplementary Table S1, #1) showed multifocal coagulative necrosis (asterisk) and lymphoid depletion (arrows; scale bar: 50 µm; HE). (**B**) The Bursa fabricii of a blackbird (Supplementary Table S1, #3) displayed marked subacute lymphocytolysis (asterisks) in follicular centers and additional histiocytic infiltrations (scale bar: 100 µm; HE). (**C**) The liver of a blackbird (Supplementary Table S1, #4) presented severe coagulation necrosis (asterisk) accompanied by mild infiltration of lymphocytes and hemosiderin-laden macrophages (scale bar: 50 µm; HE). (**D**) The heart of a blackbird (Supplementary Table S1, #3) exhibited mild to moderate, multifocal, predominantly interstitial, lymphohistiocytic myocarditis (arrows; scale bar: 50 µm; HE). (**E**) The cerebellum of a blackbird (Supplementary Table S1, #3) revealed mild, multifocal, perivascularly accentuated, lymphohistiocytic infiltrations (arrows; scale bar: 100 µm; HE). (**F**) The lung of a blackbird (Supplementary Table S1, #9) showed moderate, peribronchial, lymphohistiocytic inflammation (asterisk; scale bar: 50 µm; HE).

Using hematoxylin and eosin (HE) stained sections for histopathological examination, the majority of lesions were detected in lymphoid organs. In 95% of samples (18/19), the spleen showed multifocal coagulation necrosis in the red and white pulp with mild to marked depletion of lymphocytes in white pulp and concurrent hyperplasia of the red pulp (Fig. 2A). Macrophages contained frequently intracytoplasmic accumulations of a Turnbull blue positive, coarsely granulated brownish pigment consistent with hemosiderin (Table 2). In one animal (5%; 1/20; Supplementary Table S1, #13), lesions in the spleen were associated with a lymphohistiocytic serositis. Two USUV negative animals presented mild lymphoid depletion (67%; 2/3) and mild pigment accumulation in red pulp (33%; 1/3; Supplementary Table S1, #30). The third negative control animal (33%; 1/3; Supplementary Table S1, #28) displayed severe, multifocal necrosis within the spleen, accompanied by high numbers of intralesional bacteria. The Bursa fabricii displayed prominent subacute lymphocytolysis in follicular centers and histiocytic infiltrations in 75% (6/8) of USUV infected animals (Fig. 2B). In addition, multifocal, mild to moderate, lymphohistiocytic inflammation of pericloacal tissue was detected in all animals from which the respective localization was sampled (100%; 8/8). Bursa fabricii and cloaca were without significant findings in two negative control animals.

Liver lesions were observed in 59% (13/22) of USUV positive cases. Most animals showed severe, multifocal coagulation necrosis with sparse inflammation, characterized by mostly lymphocytes and macrophages. The liver of fewer animals (9%; 2/22) displayed necrosis of single randomly distributed hepatocytes. Less frequently (9%; 2/22; Supplementary Table S1, #4, #5), necrotic lesions were accompanied by a marked infiltration of lymphocytes and macrophages, which contained occasionally hemosiderin (Fig. 2C; Table 2). Sinusoids were frequently dilated and contained high numbers of erythrocytes and rarely microthrombi. Two animals presented several parasites, most likely trematodes, within bile ducts (9%; 2/22; Supplementary Table S1, #20, #23). Livers of USUV negative control animals showed mild, multifocal, predominantly periportal, lymphohistiocytic inflammation accompanied by hemosiderosis and single cell necrosis. Bile ducts of one control common blackbird (Supplementary Table S1, #29) contained multiple trematodes.

Lesions of the myocardium and pectoral muscles were observed in 50% (11/22) and 33% (1/3, Supplementary Table S1, #3) of USUV positive animals, respectively. Lesions were characterized by mild to moderate, multifocal, predominantly interstitial, lymphohistiocytic inflammation (Fig. 2D) which extended infrequently (14%; 3/22) into epicardium and pericardial fat tissue. Rarely, necrosis of single (cardio-)myocytes and infiltration of few heterophils was observed. Two animals showed mild, multifocal, lymphohistiocytic ganglioneuritis in subepicardial ganglia (9%; 2/22; Supplementary Table S1, #20, #26). Two of the USUV negative control blackbirds (67%; 2/3; Supplementary Table S1, #28, #30) showed a mild, focal, lymphohistiocytic, partially heterophilic myocarditis.

Brain lesions were found in 45% (10/22) of animals affecting grey and white matter of both cerebrum and cerebellum. They varied from mild to moderate, focal (23%; 5/22) or multifocal (14%; 3/22) acute necrotizing encephalitis with sparse inflammation and mild glial reactions. Three animals displayed mild, multifocal, perivascularly accentuated, lymphohistiocytic infiltrations with formation of glial nodules without necrosis within cerebrum and cerebellum (14%; 3/22; Fig. 2E). In one USUV negative control animal, a protozoal cyst without accompanying inflammation was detected focally in cerebrum (33%; 1/3; Supplementary Table S1, #28). No inflammatory lesions were observed in the brain of USUV negative blackbirds (100%; 3/3).

Histology of lungs showed lesions in 35% (8/23) of animals, which were frequently characterized by mild to moderate, peribronchial (9%; 2/23) or subpleural (17%; 4/23), lymphohistiocytic inflammation (Fig. 2F). Few animals (9%; 2/23; Supplementary Table S1, #5, #8) showed focal ulceration of bronchial epithelium and mild to moderate lymphocytolysis in adjacent bronchus-associated lymphoid tissue. In one animal, several thrombi were detected in small blood vessels (4%; 1/23; Supplementary Table S1, #1), while two other blackbirds suffered from severe, focally-extensive, subacute, necrotizing pneumonia with high numbers of intralesional fungal hyphae (9%; 2/23; Supplementary Table S1, #7, #10). In negative control animals, one blackbird showed a focal ulceration of the bronchial epithelium (33%; 1/3; Supplementary Table S1, #30) and another animal showed a focal, peribronchial, heterophilic infiltration (33%; 1/3; Supplementary Table S1, #28).

The pancreas showed mild, multifocal, randomly distributed, lymphohistiocytic inflammation with minor foci of coagulation necrosis in 33% (3/9) of USUV positive animals. A similar lesion was detected in one sample of an USUV negative blackbird (33%; 1/3; Supplementary Table S1, #29).

No significant histopathological lesions were observed in the proventriculus (100%; 9/9) and the gizzard (100%; 9/9). Moderate, multifocal, lymphohistiocytic ganglioneuritis, serositis and steatitis were detected in the intestine and the adjacent mesentery of 16% (3/19) of USUV positive blackbirds. These findings were accompanied by multifocal detection of different protozoal and helminthic parasites within intestinal lumen in almost all cases. Moreover, the same intestinal histologic lesions were observed in three negative control animals.

Kidneys were rarely affected. Only the great grey owl (6%; 1/18; Supplementary Table S1, #1) presented a mild, multifocal, lymphoplasmahistiocytic, partially heterophilic, interstitial inflammation. Kidneys of negative control blackbirds were histologically unremarkable.

### Immunohistochemistry

#### Detection of USUV antigen

Distribution of viral antigen was investigated by immunohistochemistry with an USUV specific antibody (U433). Findings are summarized in Table 1.

**Table 1:** Summarized immunohistochemical findings for the detection of Usutu virus-antigen in various organs and animals.

Organ	Number of animals positive for USUV antigen			Summarized total number of animals positive for USUV antigen	Number of animals lacking viral antigen expression	Total number of examined animals
	**Mild**	**Moderate**	**Marked**			
Spleen	9	3	4	16 (84%)	3 (16%)	19
Bursa fabricii	3	3	-	6 (75%)	2 (25%)	8
Liver	3	7	6	16 (73%)	6 (27%)	22
Heart	13	5	-	18 (82%)	4 (18%)	22
Skeletal muscle	2	-	-	2 (67%)	1 (33%)	3
Brain	10	7	2	19 (86%)	3 (14%)	22
Lung	11	8	-	19 (83%)	4 (17%)	23
Pancreas	6	1	-	7 (78%)	2 (22%)	9
Proventriculus	4	3	-	7 (78%)	2 (22%)	9
Gizzard	3	3		6 (67%)	3 (33%)	9
Intestine	6	6		12 (71%)	5 (29%)	17
Kidney	7	6	-	13 (72%)	5 (28%)	18

USUV, Usutu virus; mild, few immunopositive cells; moderate, moderate numbers of immunopositive cells; marked, high numbers of immunopositive cells; -, lacking specific score.

**Figure 3:**
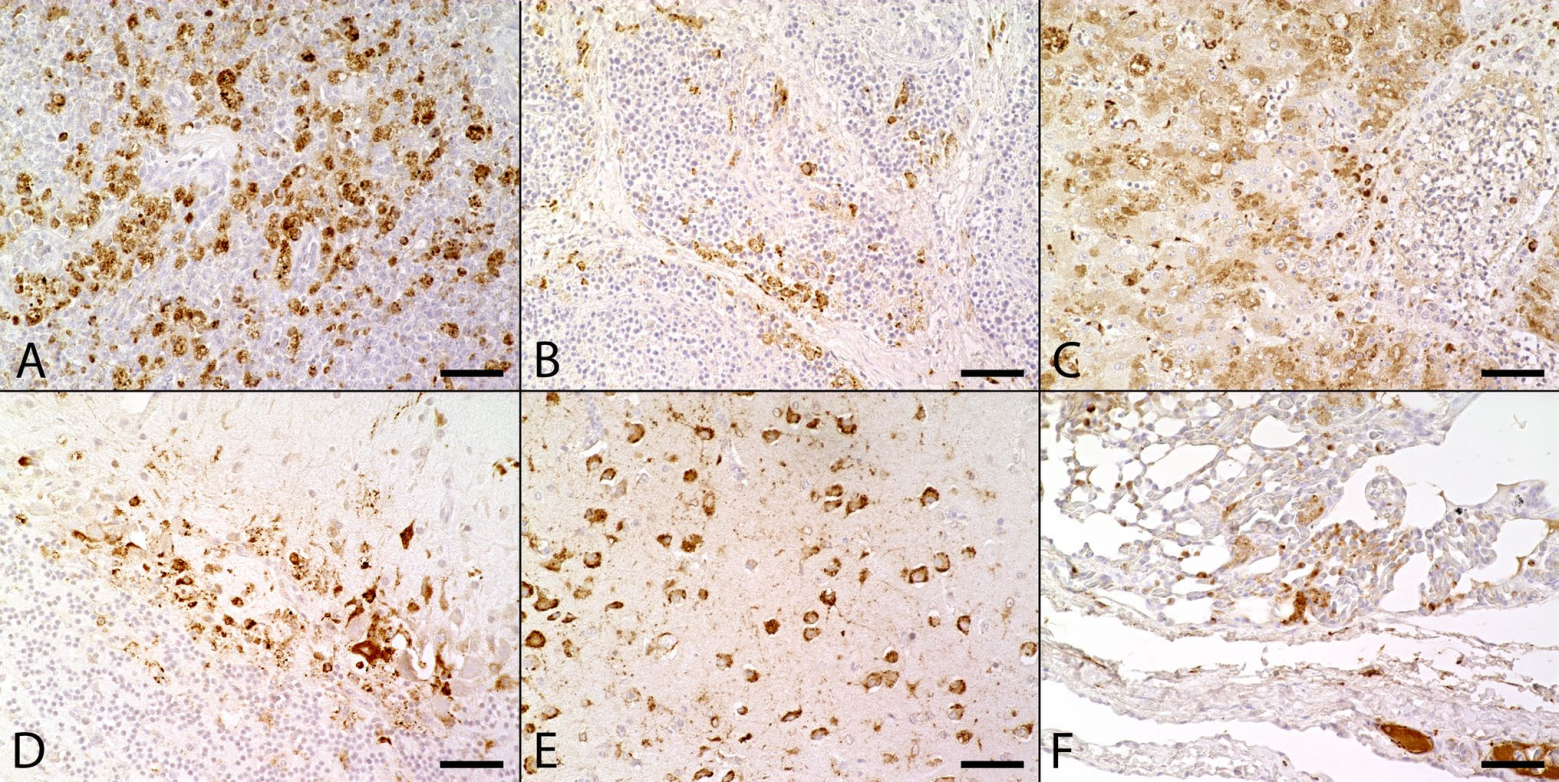
Immunohistochemical findings in naturally Usutu virus (USUV) infected birds. (**A**) USUV antigen was detected in the cytoplasm of a high numbers of cells, mostly macrophages, in both red and depleted white pulp in the spleen of a great grey owl (Supplementary Table S1, #1; scale bar: 50 µm; U433). (**B**) The Bursa fabricii of a blackbird (Supplementary Table S1, #2) showed low to moderate amounts of viral antigen, frequently located in the periphery of follicles (scale bar: 50 µm; U433). (**C**) The liver of a great grey owl (Supplementary Table S1, #1) presented moderate amounts of viral antigen multifocally in the cytoplasm of necrotic and intactly appearing hepatocytes as well as in Kupffer cells (scale bar: 50 µm; U433). (**D**) The cerebellum of a blackbird (Supplementary Table S1, #7) showed high amounts of viral antigen predominantly within Purkinje cells and glial cells (scale bar: 50 µm; U433). (**E**) In the cerebrum of a blackbird (Supplementary Table S1, #7) high numbers of unaltered neurons contained USUV antigen (scale bar: 50 µm; U433). (**F**) The lung of a great grey owl (Supplementary Table S1, #1) yielded low to moderate amounts of viral antigen in the interstitium and multifocally within lumina of small and large blood vessels (scale bar: 50 µm; U433).

In the spleen, low to high numbers of cytoplasmic positive cells were detected within the red and depleted white pulp in 84% (16/19) of USUV positive animals. Virus specific antigen was present, especially within the spleen of the great grey owl (Fig. 3A). In animals with less amounts of antigen (47%; 9/19), positive cells were often located predominantly nearby medium to large-sized blood vessels. Based on their morphology, they represented presumably macrophages. In the spleen of three animals (16%; 3/19), USUV antigen was not detected by immunohistochemistry, although animals were tested positive by qRT-PCR.

In the Bursa fabricii, low to moderate numbers of immunolabeled cells were present in 75% (6/8) of the animals. Antigen was frequently located within the cytoplasm of peripheral follicular cells or in depleted follicle centers (Fig. 3B). In addition, inflamed pericloacal connective tissue was tested positive for viral antigen in all animals investigated (100%; 8/8).

In the liver, low to high numbers of immunopositive cells with a multifocal distribution were detected within the cytoplasm of necrotic and viable hepatocytes as well as in Kupffer cells in 73% (16/22) of animals (Fig. 3C). Similarly to the spleen, abundant antigen expression was present in the liver of the great grey owl. No viral antigen was detected in liver of six animals (27%; 6/22) despite positive qRT-PCR results.

The myocardium showed mild to moderate labeling for USUV antigen in 82% (18/22) of the animals, frequently found within interstitial fibrocytes and within perivascularly infiltrating macrophages. Moreover, viral antigen was present in pericardial fat tissue and/or ganglia in animals with inflammation of the epicardium. Only mild labeling was seen within interstitium of pectoral muscles in 67% (2/3; Supplementary Table S1, #2, #3).

In the brain, low to high numbers of immunopositive glial cells were present multifocally within necrotic foci (80%; 8/10; Fig. 3D) and in the cytoplasm of neurons and Purkinje cells without cytological changes. In 55% of animals (12/22), viral antigen was predominantly located next to areas characterized by mild, multifocal, perivascular, lymphohistiocytic inflammation (Fig. 3E). Moreover, neurons without corresponding histopathologic lesions were found positive for virus antigen in 32% of cases (7/22). Three brain samples (14%; 3/22) did not show any immunopositivity for USUV antigen.

In the lung, low to moderate numbers of immunopositive cells were present in interstitial cells in 83% (19/23) of USUV positive animals (Fig. 3F). In addition, there was frequent labeling of intravascular cells (Fig. 3F). Four animals (17%; 4/23) lacked viral antigen in the lung tissue.

Low to moderate numbers of immunopositive cells were also present within the exocrine pancreas in 78% (7/9) of investigated samples, while two pancreas samples (22%; 2/9; Supplementary Table S1, #5, #14) did not show any immunolabeling.

In the gastrointestinal tract, viral antigen was frequently present in the proventriculus (78%; 7/9) and/or gizzard (67%; 6/9). Labeling yielded low to moderate amounts of viral antigen in the lamina propria mucosae, subserosal fibrous tissue, and few epithelial cells of gastric pits. Antigen was not detected in gizzard and/or proventriculus of two animals. In the intestine, mild to moderate amounts of viral antigen were found in enterocytes, crypt epithelial cells, and in the lamina propria mucosae in 71% (12/17). Less frequently, lower numbers of positive cells were found in tunica muscularis. In one animal with subserosal ganglioneuritis, moderate amounts of viral antigen were present intralesionally (6%; 1/17; Supplementary Table S1, #5). Immunolabeling was lacking in intestinal samples of five animals (29%; 5/17).

72% (13/18) of investigated kidneys yielded immunolabeling. Low to moderate amounts of viral antigen were present in interstitial cells and tubular epithelial cells of animals that lacked histological lesions (71%; 12/17). Moreover, there was a moderate labeling in the great grey owl, the only animal with interstitial nephritis. Renal tissue of five other animals (28%; 5/18) stained negative.

Throughout all investigated organs, viral antigen was most frequently present within lesions infiltrated by lymphocytes and macrophages. Based on the morphology of positively stained cells, it is suggested that the vast majority of these positive cells represent macrophages. In addition, epithelial cells were infrequently positive in a broad variety of different organs. To further identify the cell tropism of USUV, a more detailed investigation of infected cell types was performed.

### Immunohistochemical characterization of inflammatory cells

**Figure 4:**
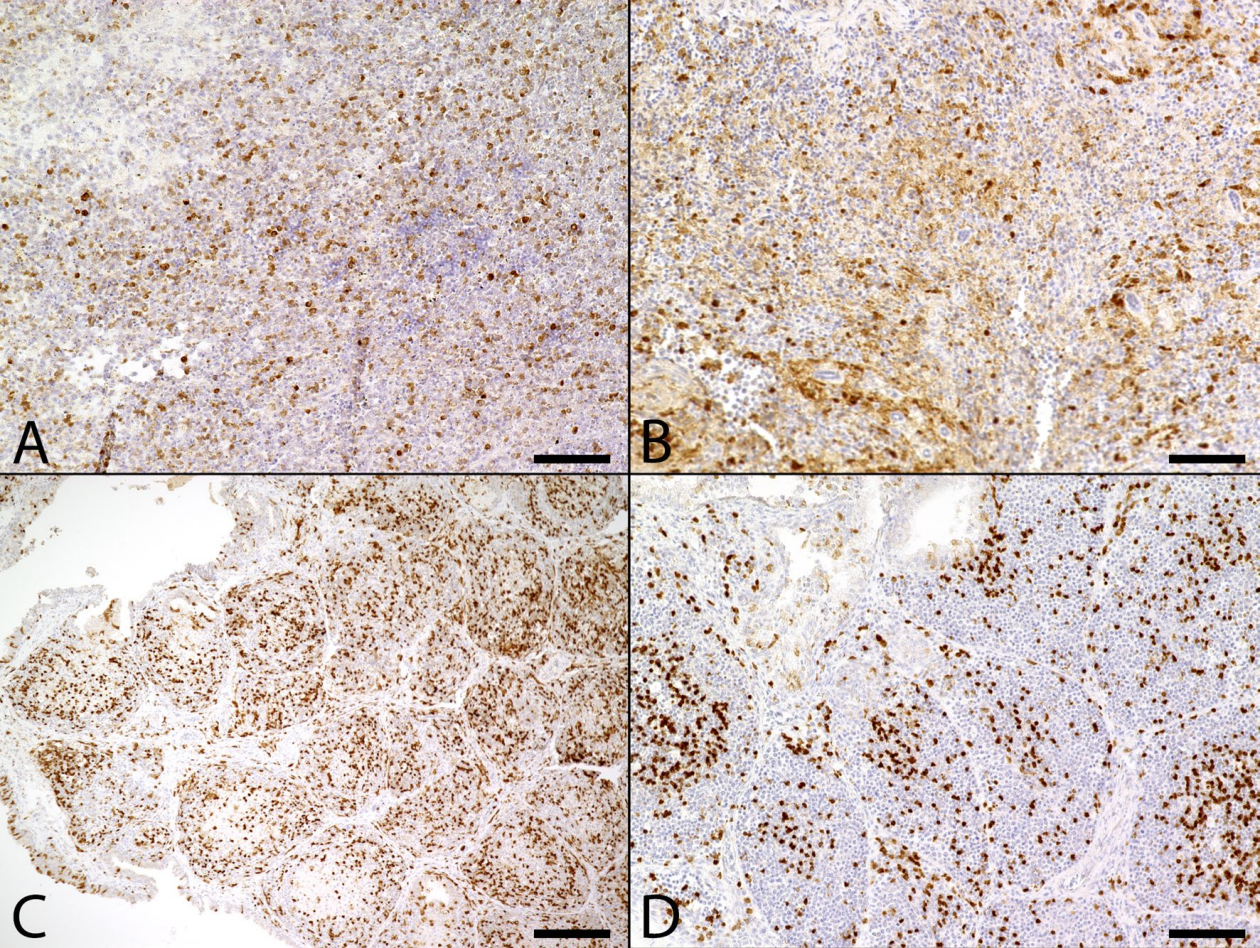
Immunohistochemical detection of Caspase 3 positive apoptotic cells in naturally Usutu virus (USUV) infected birds. (**A**) The spleen of an USUV infected blackbird (Supplementary Table S1, #3) presented mildly to moderately increased numbers of apoptotic cells (scale bar: 50 µm; Caspase 3). (**B**) The spleen of an USUV negative control blackbird (Supplementary Table S1, #29) showed less numbers of apoptotic cells. Due to the poor state of preservation, non-specific background staining was increased (scale bar: 50 µm; Caspase 3). (**C**) The Bursa fabricii of an USUV infected blackbird (Supplementary Table S1, #3) displayed a moderately increased number of apoptotic cells in follicles (scale bar: 200 µm; Caspase 3). (**D**) In the Bursa fabricii of an USUV negative blackbird (Supplementary Table S1, #29) only small to moderate numbers of apoptotic cells were detected, mostly located in the center of follicles (scale bar: 100 µm; Caspase 3).

Since most frequent and characteristic lesions in HE stained sections were found in lymphoid organs, findings were substantiated by immunohistochemical characterization of cellular components, such as T cells, B cells and apoptotic cells. In the spleen, semiquantitative analysis for T and B cells revealed mildly reduced amounts of both lymphocyte populations (92%; 12/13). Immunohistochemistry of Bursa fabricii revealed similar results. In most USUV infected birds, immunohistochemical labeling for apoptotic cells exhibited moderately increased numbers of Caspase 3 positive cells in spleen (63%; 12/19; Fig. 4A) and Bursa fabricii (75%; 6/8; Fig. 4C) of most of USUV infected animals in comparison to negative control animal (Fig. 4B,D). However, due to poor preservation state of many animals examined, unspecific background staining varied from mild to severe and interfered severely with a reliable interpretation. Immunohistochemical staining for apoptotic cells in brain tissue showed single apoptotic cells in the cerebrum and cerebellum in some animals. In most cases, these were localized at the border of white and gray matter. Unfortunately, a detailed analysis of the Caspase 3 immunohistochemistry was not possible in most sections due to high background staining most likely caused by advanced autolysis.

Identification of macrophages by immunohistochemistry using antibodies directed against CD204, Iba1, and KUL01 was performed by applying different antigen retrieval systems (no antigen retrieval, heat-induced antigen retrieval, proteolytic-induced antigen retrieval) but was not successful in blackbirds, great grey owl, and kingfisher.

### Expression of viral dsRNA

**Figure 5:**
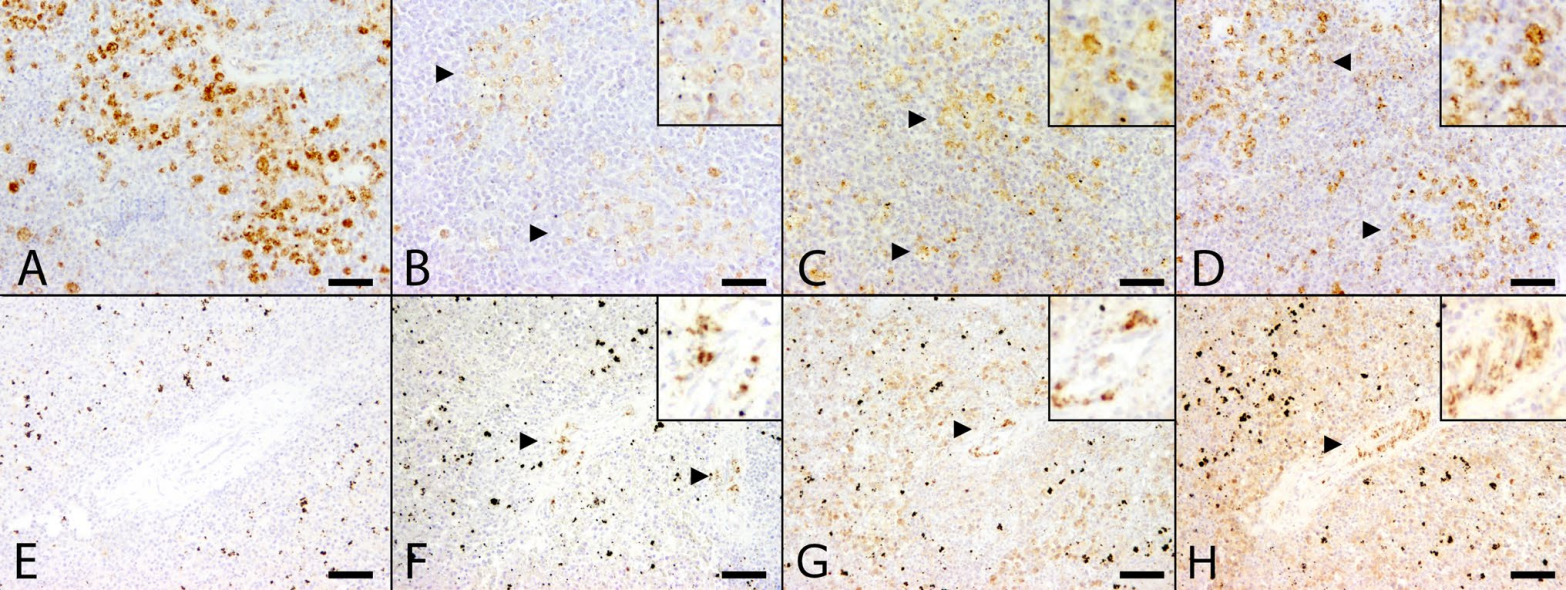
Comparison of distribution of Usutu virus (USUV) specific antigen and double-stranded ribonucleic acid (dsRNA) expression in the spleen between two animals with qRT-PCR confirmed USUV infection with (A-D) and without (E–H) immunohistochemical USUV antigen detection. (**A**) Moderate amounts of USUV antigen-labeled cells in the spleen of an animal (Table 2, #1) with USUV confirmed infection by qRT-PCR. (**B**) In contrast, only few cells labeled positive for dsRNA by applying the 9D5 antibody (arrowheads). Inset: 9D5-labeled cells at higher magnification. A moderate number of cells showed positive reaction with dsRNA-specific antibodies K1 (**C,** arrowheads) and J2 (**D,** arrowheads) similar to the USUV antigen distribution with respect to both, amount and distribution of immunopositive cells. Inset: K1- and J2-labeled cells at higher magnification. (**E**) Despite lack of viral antigen the spleen of a blackbird with qRT-PCR-confirmed USUV infection (Table 2, #7) was tested positive for dsRNA by the application of antibodies directed against dsRNA. Although, the amount of immunopositive cells varied substantially: Detection level of dsRNA by using the 9D5 antibody (**F,** arrowheads) remained low in comparison to K1 (**G,** arrowhead) and J2 (**H,** arrowhead) antibody, respectively. Insets: 9D5-, K1- and J2-labeled cells at higher magnification. (scale bar: 50 µm; immunohistochemistry).

Liver and spleen samples of six animals which tested USUV positive by qRT-PCR were further immunohistochemically investigated for dsRNA expression by using the 3 different antibodies 9D5, K1 and J2. Immunohistochemical findings were summarized in Table 2. Animals were tested negative for USUV-specific antigen expression by immunohistochemistry either in spleen and/or liver or at least in one organ despite positive qRT-PCR results. Investigation was extended by including control animals tested either positive (Table 2, #1) or negative (Table 2, #29) for USUV by both qRT-PCR and expression of virus-specific antigen. Each organ with positive immunohistochemical staining for USUV specific antigen, as shown with the antibody U433, also expressed dsRNA using J2 and K1 (Table 2, #6, #25). The same applies for the positive control animal which tested positive for USUV by both qRT-PCR and immunohistochemistry (Table 2, #1; Fig. 5A-D) as well as for two animals that lacked a positive signal for viral antigen. They revealed mild to moderate amounts of cytoplasmic immunopositive cells for dsRNA (Table 2, #7, #9). In general, positive labeling for dsRNA was co-localized with USUV antigen expression and was observed exclusively in the cytoplasm. However, the amount of immunopositive cells for the expression of dsRNA differed substantially, depending on the antibody used. Detection level of J2 and K1 ranged from mild to moderate and signaling pattern was similar compared with viral antigen distribution in liver and spleen (Table 2, #1, #6, #7, #9, #25). In contrast, immunolabeling with the 9D5 specific antibody resulted in few, weakly labeled positive cells in only two animals with confirmed USUV infection (Table 2, #1, #6). In one case, no signal was visible for dsRNA using 9D5 despite viral antigen expression (Table 2, #25).

**Table 2:** Summary of immunohistochemical and histochemical findings referring to the expression of double-stranded ribonucleic acid (dsRNA) in organs of animals lacking USUV antigen expression despite positive qRT-PCR results.

Number (#)	Species (internal ID)	USUV (qRT-PCR) result	Organ	Turnbull blue staining	Detection of USUV antigen	Expression of dsRNA		
						9D5	K1	J2
1	Great grey owl (S763/18)	+	Liver	Mild	Marked	Moderate	Marked	Marked
			Spleen	Negative	Moderate	Mild	Mild	Moderate
6	Blackbird (S904/18)	+	Liver	Moderate	Negative	Negative	Negative	Negative
			Spleen	Mild	Mild	Mild	Mild	Mild
7	Blackbird (S939/18)	+	Liver	Mild	Negative	Negative	Moderate	Mild
			Spleen	Negative	Negative	Mild	Moderate	Moderate
9	Blackbird (S1012/18)	+	Liver	Mild	Negative	Negative	Negative	Negative
			Spleen	Negative	Negative	Negative	Moderate	Moderate
14	Blackbird (594/11)	+	Liver	Negative	Negative	Negative	Negative	Negative
23	blackbird (k18/16)	+	Liver	Marked	Negative	Negative	Negative	Negative
			Spleen	Negative	Negative	Negative	Negative	Negative
25	Blackbird (K26/18)	+	Liver	Mild	Negative	Negative	Negative	Negative
			Spleen	Mild	Mild	Negative	Mild	Mild
29	Blackbird (S988/18)	−	Liver	Negative	Negative	Negative	Negative	Negative
			Spleen	Negative	Negative	Negative	Moderate	Moderate

USUV, Usutu virus; dsRNA, double-stranded ribonucleic acid; mild, few positive cells; moderate, moderate numbers of positive cells; marked, high numbers of positive cells; + , positive; -, negative.

However, only the spleen of one animal with a lack of viral antigen expression yielded positive signals for all three antibodies (Table 2, #7; Fig. 5E-H), while liver of the same animal as well as spleen of the other blackbird lacked immunopositivity for the 9D5 antibody. Furthermore, negative control displayed moderate amounts of immunopositive cells in spleen for J2 and K1 but not 9D5 (Table 2, #29). Investigation of remaining animals (Table 2, #14, #23) did not provide further findings. The disturbing, non-specific dark brown precipitate (Table 2, #7; Fig. 5E,F) occurring in numerous organ samples in both, USUV positive animals and negative controls was interpreted as unspecific reaction due to hemosiderosis and the deposition of formalin pigment. More important, few brownish decorated cells represent specific staining for dsRNA. Further investigations regarding a relationship between qRT-PCR Ct values and the expression of dsRNA as a possible explanation for lack of immunopositivity for virus specific antigen in two animals (Table 2, #7, #9) was not possible, since Ct values were not available for these animals. Ct values for 16 animals were obtained from brain tissue, therefore the investigation for a relationship of Ct values, immunohistochemical USUV antigen expression and the presence of dsRNA was performed on these animals and findings were summarized in Supplementary Table 3. Twelve animals (Supplementary Table 3, #1, #2, #4, #5, #15, #18, #21, #22, #23, #25, #26, #27) with Ct values ranging from 14.11 to 39.25 were shown to be immunopositive for USUV antigen and dsRNA in brain tissue. Furthermore, three animals (Supplementary Table 3, #17, #20, #24) with Ct values ranging from 14.82 to 29.38 tested negative for immunohistochemical expression of USUV antigen and dsRNA within brain tissue. In one case brain tissue was not available (Supplementary Table 3, #12). Two animals (Supplementary Table 3, #3, #11) were listed to provide information about the Ct values, although they were not obtained from brain tissue.

### Virus isolation

For virus isolation, liver, spleen and CNS samples of the great grey owl (Supplementary Table S1, #1) and liver and CNS samples of one blackbird (Supplementary Table S1, #3) were further processed. Both samples yielded measurable amounts of viral RNA by qRT-PCR upon inoculation in PK-15 and Vero E6 cells. The highest number of USUV genome copies per ml was found in liver tissue of the great grey owl (Supplementary Table S1, #1) after inoculation in PK-15 cells. The latter yielded higher titers than Vero cells and were chosen for further amplification of USUV stocks. Second highest titer was observed for an isolate from the same tissue grown on Vero cells, followed by virus isolated from spleen and brain, respectively. Similarly, for the blackbird (Supplementary Table S1, #3) highest virus titer was isolated from liver tissue, followed by brain, inoculated in PK-15 cells, though titers were generally lower than those of the great grey owl isolates.

### Phylogenetic analysis

**Figure 6:**
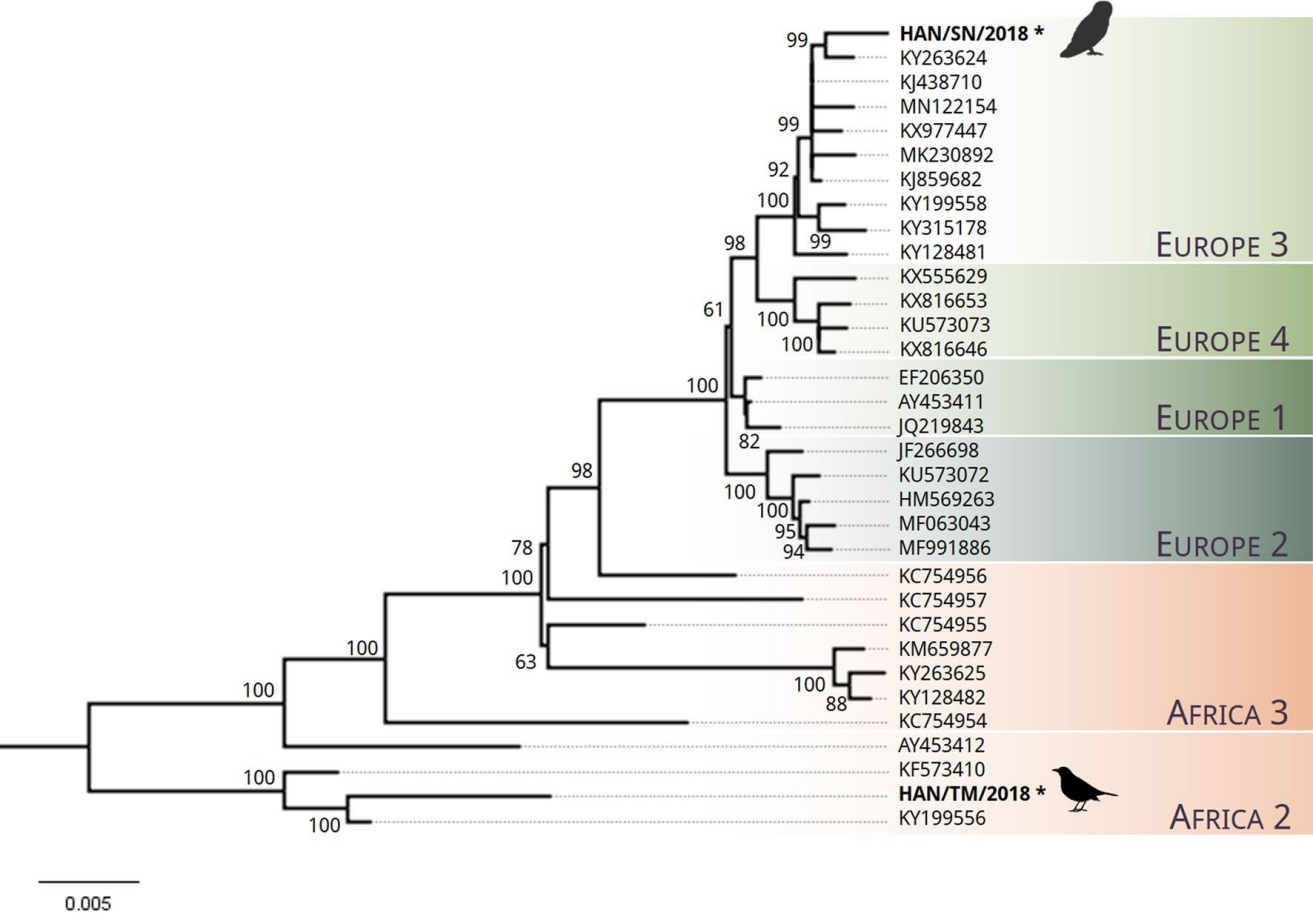
Maximum likelihood phylogeny of USUV sequences. Phylogenetic tree was constructed using a subset (n = 31) of published USUV sequences from NCBI Genbank representing USUV strains from lineages Africa 2–3 and Europe 1–4. Maximum likelihood phylogeny was performed using nearly complete genomes (> 10,203 bp) with a GTR + G model of nucleotide substitution. The tree was generated using bootstrap support of 1000 replications. Bootstrap values are depicted on the major nodes. The tip labels represent the NCBI Genbank accession numbers of respective strains and the scale bar is proportional to the number of nucleotide substitutions per site. The novel sequences of the strains “HAN/SN/2018” and “HAN/TM/2018” that were generated via NGS in this study are marked with an asterisk and were obtained from a great grey owl and a black bird, respectively. The pictogram of the owl was created with BioRender.com. The pictogram of the blackbird is licensed under CC BY-SA 3.0 (https://creativecommons.org/licenses/by-sa/3.0/legalcode) and originally created by Andreas Plank and can be found under the following link: https://commons.wikimedia.org/wiki/File:Blackbird_Turdus_merula_female_silhouette.svg.

A full-length USUV genome sequence was assembled from NGS reads generated from RNA extraction from pooled liver and brain tissues sample of the great grey owl (Supplementary Table S1, #1; HAN/SN/2018). Sequence analysis of HAN/SN/2018 showed a 99.76% sequence homology to USUV strain V19 (GenBank accession no. KJ438710) and that it is phylogenetically related to Europe 3 lineage strains (Fig. 6). A partial USUV sequence representing 92% genome coverage was also obtained from a blackbird tissue sample (Supplementary Table S1, #3; HAN/TM/2018). Phylogenetic analysis showed this sequence clusters with Africa 2 strains of USUV (Fig. 6).

## Discussion

The present investigation analyzed pathological findings in naturally USUV infected birds in Germany between 2011 and 2018, including macroscopy, histopathology, immunohistochemistry and phylogenetic analysis. It revealed that even 10 years after the first detected case in Germany, USUV is still considered a threat to the German wild and zoo bird population, especially to common blackbirds. Until today, eight different lineages of USUV have been detected, which co-circulate frequently in the same geographic area^5,12^. Virus lineage Europe 3, which was phylogenetically identified in this study, was first detected in wild birds in South Germany about 10 years ago with a spread northwards in the last years^9–11,22,30^. Whereas, the occurrence of USUV lineage Africa 2 was first reported only in the region of Berlin in 2015 and 2017 and for the second time in Saxony, region of Leipzig in 2017 and 2018^11,55^. Only one USUV lineage is assumed to be endemic throughout Germany^1^.

In this study, macroscopic lesions of USUV positive animals, most frequently characterized by hepatomegaly and splenomegaly, were largely consistent with those previously described^11,21,56^. Histopathologic changes also largely reflected observations previously described that may develop in the course of infection with USUV. However, frequency of individual lesions differed from previous studies from other European countries^21,56^. Thus, in the present study, 95% of the animals examined showed necrosis in spleen, whereas previous studies could only detect this kind of alteration in about 47% and 54% of investigated cases, respectively^21,56^. Furthermore, changes in liver, mostly characterized by necrosis, were detected in 59% of animals in this study. However, in previous publications proportion of animals showing lesions within liver varied between 44 and 63%^21,56,57^. Interestingly, histological lesions within the brain were observed in only 45% of examined USUV infected animals in this study. This is in contrast to previous reports of 69% and up to 90%, respectively^21,56^. However, investigations on USUV outbreaks in Germany show a similar distribution pattern of histopathological lesions as found in this study^11^.

Recent studies have shown that there are significant differences in the virulence of different lineages in both mouse models and in vitro cell culture experiments^28,39,58^. In particular, neurovirulence was found to be increased in the Europe 2 lineage compared to the other lineages in one study^39^. Since these experiments were performed in the mouse model^28,39,58^ on the one hand and under in-vitro conditions^39^ on the other hand, the results should be interpreted cautiously in the context of the present study.

The predictive value and transferability of results from the mouse model to birds is not sufficiently clarified and requires further research. However, it can be assumed that similar differences in USUV pathogenicity depending on the lineage can also be observed in birds, especially in blackbirds.

For virus isolation, two animals were selected based on amount of viral antigen detected by immunohistochemistry and their good preservation state. Immunohistochemical investigation for USUV antigen revealed immunolabeled cells in all tested organs of both animals. Organs that were found to be antigen positive by immunohistochemistry contained cultivable infectious virus when inoculated in permissive cell lines. Similar findings were reported in several studies^56,59,60^. However, in most studies, virus isolation was not possible in all animals examined due to poor state of preservation^9,23,56,60,61^. A direct relationship between advanced autolysis is assumed in some of these cases^9^. Systemic USUV replication in these bird species has previously been observed in combination with virus detection in many organs such as liver, heart, brain, and spleen^9,21^.

A previous study described presence of viral antigen and RNA predominantly in brain^21^. There, viral antigen could be detected by immunohistochemistry in each brain examined from animals that were shown to be infected by applying qRT-PCR. However, viral antigen could only be visualized immunohistochemically in the brain in 80% of present cases. Moreover, considerable amounts of viral antigen were detected in 84% of splenic specimens. Overall, detection of viral antigen and infectious virus in liver and viral antigen in spleen is not surprising, as several studies have already shown that USUV not only exhibits a neurotropism, but rather a pantropism^9,11,59,62^.

Further clarification of viral tropism in inflammatory cells was attempted by use of commercially available antibodies, designated for use in other avian species, by immunohistochemistry or immunofluorescence dual staining. Unfortunately, this approach failed since macrophages of blackbirds were not detectable on formalin-fixed and paraffin-embedded tissue by any of the used markers.

In addition, in one case, viral antigen could not be detected in any analyzed organ by immunohistochemistry, although this animal was shown to be infected with USUV. This phenomenon is most likely to be interpreted as a consequence of poor state of preservation of this carcass at the time of sampling. However, it also could indicate lack of sensitivity of applied antibody, early phase of infection with reduced virus translation or restricted virus infection. In this regard, it has been shown for WNV infected birds, that the time frame for the detection of viral antigen in tissue samples is closely associated with severe clinical disease^63^. As anamnestic data of the birds included in the present investigation are not available, a determination of the time point of infection is not possible, the histopathological examination indicated a subacute to chronic infection.

To further investigate this phenomenon, an additional different methodological approach was employed. More precisely, we investigated the expression of dsRNA as an early indicator of viral infections in the aforementioned cases. Based on highest detectable levels of USUV genome copies as well as sufficient viral antigen expression we concentrated our investigations for dsRNA on liver and spleen. As expected, liver and spleen samples, which tested positive for USUV antigen by immunohistochemistry and qRT-PCR, displayed positive results by the investigation for dsRNA. Unexpectedly, detection level of different antibodies directed against dsRNA differed substantially, although it is assumed that all applied antibodies recognize dsRNA with a length of at least 30 base pairs (bp), independently of sequence^41^. In the present study, especially 9D5 seemed to detect dsRNA to a lesser extent in comparison to J2 and K1. In addition, antibodies J2 and K1 exhibited a positive reaction in spleen tissue of negative control animal. This could be interpreted as false positive results. Furthermore, it still remains an option that an additional viral infection, which was not detected, could be the cause for this reaction. Another important factor, which has to be taken into consideration, represents endogenous dsRNA, which occurs as a result of enzymatical processing of premature microRNAs (pre-miRNAs) with a length of approximately 70 bp into mature miRNAs comprised of 20 bp^64^. On one hand, the investigation on the relationship of qRT-PCR data, dsRNA and viral antigen detection revealed in 66% of animals with available Ct values a possible correlation between these parameters. Ct values up to 23.82 were accompanied by simultaneous presence of viral antigen and dsRNA. Higher Ct values (greater than 24.79) of investigated animals were not associated with either USUV antigen expression or the presence of dsRNA. On the other hand, 27% of animals, in which Ct values obtained from brain tissue ranged from 29.42 to 39.25, were tested positive for USUV antigen and dsRNA by immunohistochemistry. In contrast, Ct values lower than 14.82 were not concomitant with USUV antigen expression and immunopositivity for dsRNA in 7% of birds. The observed discrepancies could be explained by advanced autolysis. Still, presence of dsRNA in USUV positive animals, despite lack of viral antigen, indicates suitability of dsRNA as a sensing tool for early virus infection^41^, in which viral antigen is possibly not yet detectable. As shown before with WNV, the time frame for the detection of viral antigen is small and closely associated with clinical disease^63^. Additionally, detection of dsRNA and absence of viral antigen at the same time might indicate a higher sensitivity of dsRNA specific antibodies for viral dsRNA in contrast to virus specific antibodies. Furthermore, divergence in detection level of qRT-PCR in comparison to virus specific antibody immunoreactivity was already described by others^65^: It is assumed that numerous infected cells with minor viral load are rather positive in PCR than single cells with high viral loads.

In summary, this study showed that detection of USUV antigen is not necessarily associated with histopathological lesions in corresponding organs, regardless of the virus lineage. Although detection of dsRNA can be useful to demonstrate virus infections, dsRNA positive reactions in birds should be interpreted with great caution as in any other species. Therefore, further investigations are needed to evaluate the applicability of dsRNA as an alternative method for virus discovery, especially in avian species in naturally occurring virus infections.

## Materials and methods

### Animals and tissue samples

This study investigated 27 birds which were tested positive in different tissue samples for USUV and negative for WNV by quantitative real-time PCR (qRT-PCR) at the Friedrich-Loeffler-Institut (FLI), Riems, Germany. In detail, 25 common blackbirds, one great grey owl, and one kingfisher, collected over a period from 2011 to 2018, were assessed (Supplementary Table S1). Noteworthy, not all tissues were available from all animals. Taken together, samples from lungs (n = 23), brains (n = 22), hearts (n = 22), livers (n = 22), spleens (n = 19), kidneys (n = 18), intestines (n = 17), stomachs (gizzards and/or proventriculus; n = 12), pancreases (n = 9), Bursa fabricii with cloaca (n = 8), and pectoral muscles (n = 3) were investigated. Heterogeneity of collected samples is related to varying sampling protocols at different institutions as well as advanced autolysis and/or tissue loss due to post mortem predation in some of the cases. In addition, tissue samples from two animals which tested negative for USUV by qRT-PCR were included as negative controls.

This study was carried out in accordance to the German animal welfare act. All animals were dead at the time of submission for necropsy and post-mortem examinations were carried out to investigate the causes of illness or death.

### Quantitative real-time PCR (qRT-PCR)

Detection of USUV by qRT-PCR was performed as previously described^1,11^. Briefly, RNA was extracted from organ samples using RNeasy Mini Kit (Qiagen, Hilden, Germany) according to manufacturer’s protocol. Extracted RNA was analyzed by amplification of an USUV specific 91 nucleotide long region of the nonstructural protein 1 gene^8^. In addition, an infection with WNV was excluded by qRT-PCR with specific 5’NTR-region primers^66^.

### Virus isolation

One day prior to the virus isolation, Vero E6 and PK-15 cells were seeded at 2.5 × 10^5^ cells/ml in 6-well plates. Approximately 50 mg tissue of central nervous system (CNS), liver and spleen of an infected great grey owl (Supplementary Table S1, #1) as well as CNS and liver of a blackbird (Supplementary Table S1, #3) were cut into small pieces^1^. Thereafter, 500 µl sterile phosphate-buffered saline (PBS) was added to the tissue before homogenization for 40 s in a tissue lyzer (Fastprep, MP Biomedicals). The homogenate was centrifuged for one minute at 17,000 × g. The supernatant was transferred into a filter tube and centrifuged for one minute. The filtered supernatant was mixed with 1 ml Dulbecco´s Modified Eagle Medium [DMEM, admixed with 2% fetal calve serum (FCS), 1% penicillin/streptomycin and 1% gentamicin]. Hereof, 500 µl each were used to infect Vero E6 and PK-15 cells, respectively. Cells were inoculated for one hour before they were transferred into 2 ml DMEM (admixed with 2% FCS, 1% penicillin/streptomycin and 1% gentamicin). Three days post infection, supernatants of infected cells were used for viral RNA extraction (Qiagen viral RNA mini kit) and qRT-PCR to determine viral genome copy numbers^8^.

### Histopathology and immunohistochemistry

During necropsy, tissue samples were fixed in 10% neutral buffered formalin for at least 24 h. Thereafter, specimens were trimmed, embedded in paraffin wax and routinely stained with HE for analysis by routine light microscopy. Additionally, Turnbull blue staining was performed on selected sections for visualization of iron deposits (hemosiderin).

Immunohistochemistry was used to detect USUV antigen by the polyclonal antibody U433 which detects in Germany circulating lineages of USUV, including Europe 3, Africa 2 and 3 (kindly provided by FLI, Germany^11^), T lymphocytes (CD3, DakoCytomation, Germany), B lymphocytes (CD268, BIO-RAD, Germany), and apoptotic cells (Caspase 3, Cell Signaling, USA) as well as dsRNA [J2 (SCICONS, Hungary), K1 (SCICONS, Hungary) and 9D5 (Absolute Antibody, UK)]. For identification of macrophages antibodies directed against CD204 (Transgenic Inc., Japan), Iba1 (Thermo Fisher, USA) and KUL01 (BIO-RAD, Germany) were applied. Used primary antibodies, dilutions, and pre-treatments are summarized in Supplementary Table S2. After de-paraffinization and rehydration, endogenous peroxidase activity was blocked using 85% ethanol with 0.5% hydrogen peroxide for 30 min at room temperature (RT). Heat-induced epitope retrieval was performed for all antibodies except for 9D5, in respective buffer solution in the microwave (600 W) for 20 min (Supplementary Table S2). For 9D5, proteolytic-induced epitope retrieval was achieved by incubation with Proteinase K [1000 ml PBS admixed with 3 µl Proteinase K (Roche Diagnostics, Germany)] for 7 min. Thereafter, sections were blocked with inactivated normal goat serum (diluted 1:5 in PBS, pH 7.2) for 30 min to prevent unspecific binding. Primary antibodies, diluted in PBS/bovine serum albumin (BSA), were applied in their respective concentration (Supplementary Table S2) followed by incubation for 90 min at RT. For negative controls, primary antibody was replaced by normal rabbit serum (diluted 1:3000 in PBS) or Balb/c serum (diluted 1:1000 in PBS). After subsequent washing with PBS, sections were incubated for 60 min at RT with secondary biotinylated goat-anti-rabbit or goat-anti-mouse antibody (diluted 1:200 in PBS), respectively. Incubation was followed by application of avidin–biotin-peroxidase complex (Vectastain ABC Kit Standard, Vector Laboratories, USA) according to manufacturer’s protocol. Visualization of reaction was achieved using 3,3-diaminobenzidine tetrahydrochloride (0.05%, Sigma Aldrich Chemie GmbH, Germany) with addition of 0.03% hydrogen peroxide. Finally, sections were counterstained with Mayer’s hematoxylin (Roth C. GmbH & Co KG, Germany).

For investigation of viral dsRNA, immunohistochemistry was performed using Dako EnVision + polymer system (Dako North America, USA) according to manufacturer´s protocol. Counterstaining was performed as described above.

### Next generation sequencing (NGS)

Two USUV positive birds, one great grey owl and one blackbird from Northern Germany (Supplementary Table S1, #1, #3), were selected for NGS based on the amount of viral antigen detected by immunohistochemistry and preservation state. Cerebrum and liver tissue samples from each animal were pooled in PBS and homogenized using ceramic beads in a FastPrep-24 5G homogenizer (MP Biomedical). Prior to library preparation, viral genome enrichment strategies were used as previously described^67^. Briefly, the homogenized tissues of the two birds were exposed to three freeze/thaw cycles and filtered through 0.45 µm Ultrafree-MC Spinfilters (Merck Millipore) to release viral particles and minimize host cellular debris. RNA extraction was performed using TRIzol™ (Thermo Fischer Scientifics, USA) according to manufacturer’s protocol. Reverse transcription was preformed using SuperScript IV (Thermo Fischer Scientifics, USA) in combination with a mix of non-ribosomal and random hexamers^68^. For dsDNA generation, Klenow fragment (New England Biolab) was used, followed by a random amplification step in accordance to a sequence-independent, single-primer amplification protocol as described previously^69^. The purified amplification products were used for DNA library preparation using the NextSeq 500/550 Mid Output Kit v2 kit (Illumina) following manufacturer’s instructions and sequenced with an Illumina NextSeq 550 sequencer using 300 cycles. Quality and adapter trimming, as well as reference assembly of the generated FASTQ data was performed using the CLC Genomics Workbench, v12 (Qiagen).

### Maximum likelihood tree construction

Phylogenetic analysis of USUV sequences generated in this study “HAN/SN/2018” and “HAN/TM/2018” was performed using 31 USUV sequences downloaded from NCBI GenBank. These sequences were all over 10,203 bp and included representative USUV strains from lineages Africa 2–3 and Europe 1–4. The Africa 1 lineage was omitted in order to increase the resolution of the phylogram and to focus on the most recent phylogenetic lineages. This way of phylogenetic presentation of Usutu virus strains has previously been done by other groups, too^70,71^. Europe 5 was additionally not included in the phylogenetic analysis. To our knowledge, there are no complete genomes of Europe 5 lineages published on NCBI Genbank. Sequences were aligned using the MAFFT tool (version 7)^72^ and maximum likelihood phylogeny was built using MEGA X^73^ with a GTR + G model of nucleotide substitution and bootstrap support of 1000 replications.

## Supplementary Information

Supplementary Information.
