# Supplementary material for: Analysis of avian Usutu virus infections in Germany from 2011 to 2018 with focus on dsRNA detection to demonstrate viral infections
Source: Sci Rep. 2021 Dec 17;11:24191. doi: 10.1038/s41598-021-03638-5 (PMC8683490; doi:10.1038/s41598-021-03638-5)
Supplement: Supplementary file 1 — Supplementary Information. [file 41598_2021_3638_MOESM1_ESM.docx]

**Supplementary Table S1. List of animals investigated in the present study including species, geographic origin, internal identification number (ID), anamnesis, macroscopic findings, organs available for light microscopy and quantitative realt-time PCR (qRT-PCR) analysis.**

| number (#) | species | geographic origin  (year, city/ state) | internal ID | anamnesis | macroscopic findings | organs for light microscopy | USUV (qRT-PCR) result** |
| --- | --- | --- | --- | --- | --- | --- | --- |
| 1 | great grey owl | 2018,  Hanover/ LS | S763/18 | apathetic, died one day after onset of clinical signs | hyperemia in liver and lung; dry content in ceca; subcutaneous edema | *, pancreas, pectoral muscle | + |
| 2 | blackbird | 2018,  Hanover/ LS | S823/18 | apathetic, died one day after onset of clinical signs | splenomegaly; hyperemia in liver and lung; slimy content in intestine | *, Bursa fabricii, pancreas, pectoral muscle | + |
| 3 | blackbird | 2018,  Hanover/ LS | S837/18 | found dead | splenomegaly; hyperemia in liver, lung and kidney; subcutaneous edema | *, Bursa fabricii, pancreas, pectoral muscle | + |
| 4 | blackbird | 2018,  Saarland | K29/18 | found dead | splenomegaly; serous atrophy of fat tissue | *, no spleen available | + |
| 5 | blackbird | 2017,  NRW | S1148/17 | found dead | poor nutritional condition; splenomegaly and hepatomegaly; severe intestinal nematode infestation | *, Bursa fabricii, pancreas | + |
| 6 | blackbird | 2018,  NRW | S904/18 | found dead | moderate nutritional condition; splenomegaly; cherry pits in stomach; severe intestinal tape worm infestation | brain, lung, heart, liver, spleen, stomach, intestine | + |

**Supplementary Table S1 (continued)**

| number (#) | species | geographic origin  (year, city/ state) | internal ID | anamnesis | macroscopic findings | organs for light microscopy | USUV (qRT-PCR) result** |
| --- | --- | --- | --- | --- | --- | --- | --- |
| 7 | blackbird | 2018,  NRW | S939/18 | found dead | moderate nutritional condition; pyogranulomatous aerosacculitis | *, pancreas, Bursa fabricii | + |
| 8 | blackbird | 2018,  NRW | S951/18 | found dead | moderate nutritional condition; splenomegaly and hepatomegaly; hemorrhagic enteritis; intestinal ascarids | *, pancreas, Bursa fabricii, no stomach available | + |
| 9 | blackbird | 2018,  NRW | S1012/18 | found dead | poor nutritional condition; splenomegaly and hepatomegaly | brain, lung, liver, spleen, Bursa fabricii, intestine | + |
| 10 | blackbird | 2018,  NRW | S1025/18 | found dead | poor nutritional condition; shaggy plumage; splenomegaly and hepatomegaly | * | + |
| 11 | blackbird | 2011,  Frankfurt/ Hesse | ED-I-17/11 | found dead | no gross examination performed | liver, heart, lung, spleen, kidney | + |
| 12 | blackbird | 2011,  Karlsruhe/ BW | ED-I-31/11 | found dead | no gross examination performed | liver, spleen | + |
| 13 | blackbird | 2011,  Kirchheim/ Hesse | 565/11 | found dead | no gross examination performed | spleen, heart | + |
| 14 | blackbird | 2011,  Alzey/ RP | 594/11 | found dead | no gross examination performed | brain, liver, heart, lung, kidney, pancreas, intestine | + |

**Supplementary Table S1 (continued)**

| number (#) | species | geographic origin  (year, state/city) | internal ID | anamnesis | macroscopic findings | organs for light microscopy | USUV (qRT-PCR) result** |
| --- | --- | --- | --- | --- | --- | --- | --- |
| 15 | kingfisher | 2011,  Oberelbert/ RP | ED-I-62/11 | found dead | no gross examination performed | brain, liver, lung | + |
| 16 | blackbird | 2011, Ochtendung/ RP | 595/11 | found dead | no gross examination performed | brain, liver, heart, lung, kidney, intestine | + |
| 17 | blackbird | 2011,  Hofheim/ Hesse | ED-I-85/11 | found dead | no gross examination performed | brain | + |
| 18 | blackbird | 2011,  Hofheim/ Hesse | ED-I-86/11 | found dead | no gross examination performed | brain, liver, heart, lung, spleen | + |
| 19 | blackbird | 2011,  Mainz/ RP | 588/11 | found dead | no gross examination performed | heart, lung, kidney | + |
| 20 | blackbird | 2017,  Viersen/ NRW | K3/17 | found dead | splenomegaly | brain, liver, heart, lung, spleen | + |
| 21 | blackbird | 2017, Mönchenglad-bach/ NRW | K74/17 | trauma | subcutaneous hemorrhages; empty stomach | brain, intestine, stomach | + |
| 22 | blackbird | 2017,  Erkelenz/ NRW | K75/17 | found dead | no significant macroscopic lesions | *, pancreas | + |
| 23 | blackbird | 2016, Mönchenglad-bach/ NRW | K18/16 | found dead | no gross examination performed | liver, heart, lung, spleen, kidney | + |

**Supplementary Table S1 (continued)**

| number (#) | species | geographic origin  (year, state/city) | internal ID | anamnesis | macroscopic findings | organs for light microscopy | USUV (qRT-PCR) result** |
| --- | --- | --- | --- | --- | --- | --- | --- |
| 24 | blackbird | 2013, Weiskirchen/ Saarland | K28/13 | found dead | no gross examination performed | brain, heart, lung, kidney | + |
| 25 | blackbird | 2018,  Saarland | K26/18 | found dead | splenomegaly | *, no stomach available | + |
| 26 | blackbird | 2018,  Saarland | K27/18 | found dead | splenomegaly; empty gastrointestinal tract | *, Bursa fabricii | + |
| 27 | blackbird | 2018,  Hassel/ LS | K28/18 | found dead | hyperemia in all organs | * | + |
| 28 | blackbird | 2017,  NRW | S1147/17 | found dead | poor nutritional condition; splenomegaly and hepatomegaly; severe intestinal nematode infestation | *, pancreas | - |
| 29 | blackbird | 2018,  NRW | S988/18 | found dead | no significant macroscopic lesions | *, pancreas, Bursa fabricii, no stomach available | - |
| 30 | blackbird | 2018,  NRW | S989/18 | found dead | poor nutritional condition; mild hepatomegaly; severe intestinal tape worm infestation | *, pancreas | - |

USUV, Usutu virus; LS, Lower Saxony; NRW, North Rhine-Westphalia; RP, Rhineland-Palatinate; *, brain, lung, heart, liver, spleen, kidney, gizzard and/or glandular stomach, small and large intestine; +, positive; -, negative; **, respective cycle threshold values found in Supplementary Table 3

**Supplementary Table S2. Summarized information about investigated antigen, target structure, antibody clonality, clone and species used for antibody generation, antibody dilution, used antigen retrieval method, applied secondary antibody and source of antibodies used for immunohistochemistry.**

| **antigen** | **target structure** | **clonality, clone, species** | **dilution** | **antigen retrieval** | **secondary antibody** | **source** |
| --- | --- | --- | --- | --- | --- | --- |
| **U433** | USUV antigen | pc, rabbit | 1:8000 | citrate buffer and microwave | GAR | FLI |
| **CD3** | T cells | pc, rabbit | 1:500 | citrate buffer and microwave | GAR | Dako-Cytomation |
| **CD268α** | B cells | mc, 2C4, mouse | 1:100 | citrate buffer and microwave | GAM | BIO-RAD |
| **CD204** | macro-phages | mc, SRA-E5, mouse | 1:500  1:1000 | application of different antigen retrieval systems | GAM | Transgenic Inc. |
| **Iba1** | macro-phages | pc, rabbit | 1:100  1:200  1:400 | application of different antigen retrieval systems | GAR | Thermo Fisher |
| **KUL01** | macro-phages | mc, KUL01, mouse | 1:100  1:250  1:500 | application of different antigen retrieval systems | GAM | BIO-RAD |
| **Caspase 3** | apoptotic cells | pc, rabbit | 1:500 | - | GAR | Cell Signaling |
| **J2** | dsRNA | mc, J2, mouse | 1:300 | citrate-EDTA buffer and microwave | EnVision+ System,  anti- mouse | SCICON |
| **K1** | dsRNA | mc, K1, mouse | 1:150 | citrate buffer and microwave | EnVision+ System,  anti-mouse | SCICON |
| **9D5** | dsRNA | mc, 9D5, mouse | 1:100 | Proteinase K | EnVision+ System,  anti-mouse | Absolute antibody |

pc, polyclonal; mc, monoclonal; GAR, goat anti-rabbit Immunoglobulin G (IgG); GAM, goat anti-mouse IgG; dsRNA, double-stranded ribonucleic acid; EDTA, ethylendiamintetraacetate; FLI, Friedrich-Loeffler-Institut, Greifswald-Insel Riems, Germany; -, no application

**Supplementary Table S3. Relationship between available quantitative real-time PCR cycle threshold (qRT-PCR Ct) values, immunohistochemical expression of Usutu virus antigen and the expression of double-stranded ribonucleic acid (dsRNA) in selected animals.**

| number (#) | species | internal ID | | qRT-PCR Ct value | | USUV antigen expression | dsRNA expression |
| --- | --- | --- | --- | --- | --- | --- | --- |
| 1 | great grey owl | S763/18 | | brain: 14.11 | | mild | mild |
|  |  |  |  | liver: 10.95 | | severe | moderate |
|  |  |  |  | spleen: 11.31 | | moderate | mild |
| 2 | blackbird | S823/18 | | brain: 15.28 | | mild | mild |
|  |  |  |  | spleen: 12.13 | | mild | n. d. |
| 3 | blackbird | S837/18 | | spleen: 33.49 | | mild | n. d. |
|  |  |  |  | liver: 30.99 | | moderate | n. d. |
| 4 | blackbird | | K29/18 | | brain: 18.35 | moderate | moderate |
|  |  |  |  |  | liver: 16.69 | severe | n. d. |
| 5 | blackbird | S1148/17 | | brain: 39.25 | | moderate | moderate |
|  |  |  |  | liver: 38.48 | | mild to moderate | n. d. |
|  |  |  |  | lung: 21.53 | | moderate | n. d. |
|  |  |  |  | spleen: 17.69 | | mild | n. d. |
| 11 | blackbird | ED-I-17/11 | | spleen: 17.29 | | severe | n. d. |
|  |  |  |  | liver: 21.13 | | severe | n. d. |
| 12 | blackbird | ED-I-31/11 | | brain: 23.83 | | no tissue available | no tissue available |
|  |  |  |  | spleen: 25.68 | | moderate | n. d. |
| 15 | kingfisher | ED-I-62/11 | | brain: 31.48 | | mild | mild |
|  |  |  |  | spleen: 32.66 | | no tissue available | no tissue available |
| 17 | blackbird | ED-I-85/11 | | brain: 29.38 | | - | - |
|  |  |  |  | spleen: 24.2 | | no tissue available | no tissue available |
| 18 | blackbird | ED-I-86/11 | | brain: 23.43 | | mild | mild |
|  |  |  |  | spleen: 17.56 | | severe | n. d. |

**Supplementary Table S3 (continued)**

| number (#) | species | internal ID | qRT-PCR Ct value | USUV antigen expression | dsRNA expression |
| --- | --- | --- | --- | --- | --- |
| 20 | blackbird | K3/17 | brain: 24.79 | - | - |
|  |  |  | spleen: 22.66 | moderate | n. d. |
|  |  |  | liver: 20.00 | moderate | n. d. |
| 21 | blackbird | K74/17 | brain: 23.82 | mild | mild |
|  |  |  | spleen: 26.63 | no tissue available | no tissue available |
| 22 | blackbird | K75/17 | brain: 20.07 | mild | mild |
|  |  |  | spleen: 26.63 | mild | n. d. |
| 23 | blackbird | K18/16 | brain: 29.42 | moderate | mild |
| 24 | blackbird | K28/13 | brain: 14.82 | - | - |
|  |  |  | spleen: 23.25 | no tissue available | no tissue available |
| 25 | blackbird | K26/18 | brain: 17.38 | moderate | moderate |
|  |  |  | liver: 24.17 | - | - |
| 26 | blackbird | K27/18 | brain 17.21 | moderate | moderate |
|  |  |  | liver 15.32 | moderate | n. d. |
| 27 | blackbird | K28/18 | brain: 29.56 | mild | mild |
|  |  |  | liver: 34.48 | mild | n. d. |

n. d., not done; mild, few immunopositive cells; moderate, moderate numbers of immunopositive cells; severe, high numbers of immunopositive cells; -, lack of detection
